# The role of primary pharmacological therapy in acromegaly

**DOI:** 10.1007/s11102-013-0530-0

**Published:** 2013-10-29

**Authors:** Ana Laura Espinosa de los Monteros, Carmen A. Carrasco, Alfredo Adolfo Reza Albarrán, Mônica Gadelha, Alin Abreu, Moisés Mercado

**Affiliations:** 1 Servicio de Endocrinología, Hospital de Especialidades, Centro Médico Nacional S.XXI, IMSS, Mexico City, Mexico; 2 Endocrinology Department, Facultad de Medicina, Pontificia Universidad Católica de Chile, Santiago, Chile; 3 Endocrinology and Metabolism Department, Instituto Nacional de Ciencias Médicas y Nutrición Salvador Zubirán, Mexico City, Mexico; 4 Endocrinology Section, Hospital Universitário Clementino Fraga Filho, Federal University of Rio de Janeiro, Rio de Janeiro, Brazil; 5 Endocrinology Department, Hospital Imbanaco, Cali, Colombia; 6 Faculty of Medicine, Universidad Nacional Autónoma de México, Mexico City, Mexico; 7 Endocrine Service, and Experimental Endocrinology Unit, Hospital de Especialidades, Centro Médico Nacional Siglo XXI, Instituto Mexicano del Seguro Social, MD Aristóteles 68 Polanco, 11560 Mexico City, Mexico

**Keywords:** Acromegaly, Pituitary, Latin America, GH, IGF-1, Somatostatin analogs, Dopamine agonists, Pegvisomant

## Abstract

**Background and objectives:**

Primary pharmacological therapy may be the only viable treatment option for many patients with acromegaly, especially those presenting with advanced disease with large inoperable tumors. Long-acting somatostatin analogs are currently the first-line treatment of choice in this setting, where they provide biochemical control and reduce tumor size in a significant proportion of patients. We herein present a brief overview of the role of primary pharmacological therapy in the treatment of acromegaly within the context of Latin America and support this with a representative case study.

**Case description:**

A 20 year old male presented with clinical and biochemical evidence of acromegaly. The glucose-suppressed growth hormone (GH) was 5.3 μg/L, his insulin-like growth factor-1(IGF-1) was 3.5 times the ULN and serum prolactin greater than 4,000 μg/L. Pituitary MRI revealed a large and invasive mass, extending superiorly into the optic chiasm and laterally into the left cavernous sinus. He was treated with a combination of octreotide and cabergoline with remarkable clinical improvement, normalization of GH and IGF-1 values and striking shrinkage of the adenoma.

**Conclusion:**

This case illustrates how effective the pharmacological therapy of acromegaly can be and yet at the same time, raises several important issues such as the need for life-long treatment with costly medications such as the somatostatin analogs. Access to these agents may be limited in regions where resources are restricted and clinicians face challenges in order to make the most efficient use of available options.

## Introduction

Acromegaly is characterized by excessive secretion of growth hormone (GH), usually due to a pituitary tumor, and a subsequent increase in insulin-like growth factor-1 (IGF-1) release from the liver and other tissues [1]. Long-term exposure to elevated levels of these two hormones underlies many of the debilitating clinical features of acromegaly, such as arthropathy, cardiomyopathy, diabetes mellitus and sleep apnea [1]. Pharmacological intervention in acromegaly is therefore aimed primarily at suppressing secretion of GH from the pituitary or blocking the actions of GH at it receptors, along with a secondary reduction in circulating IGF-1 levels [1, 2].

**Fig. 1:**
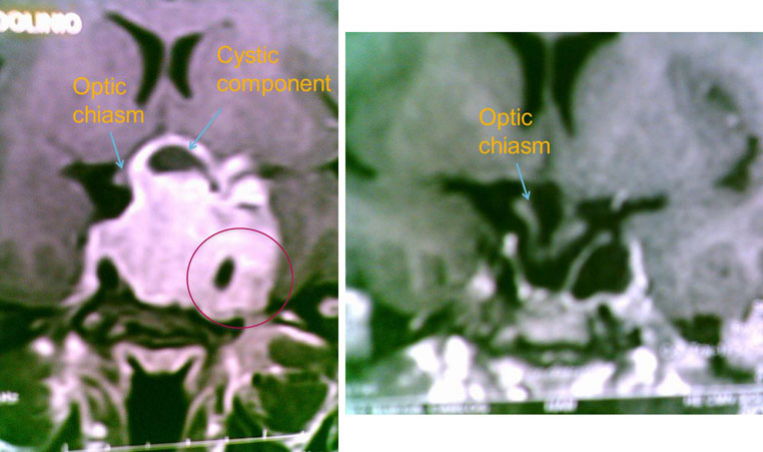
Gadolinium-enhanced, T1-weighted MRI scans (coronal view) of pituitary tumor at diagnosis (*left*) and after 6 months of treatment with an SSA and dopamine agonist

There are three classes of pharmacological therapy used routinely to control disease activity in acromegaly: dopamine agonists (principally cabergoline) and somatostatin analogs (SSAs), which suppress the secretion of GH, and a GH receptor antagonist (pegvisomant) [2]. However, access to these different classes of drugs may vary at the local level, both between and within individual countries, and this is particularly applicable to Latin America [3]. Although pharmacological therapy is used predominantly as secondary therapy for persistent or recurrent acromegaly following non curative surgery, it is increasingly being used as primary therapy in the following situations: (1) patients in whom surgery is not an initial option due to tumor location and limited chance of surgical cure, (2) as short-term therapy before surgery aimed at reducing perioperative anesthetic morbidity, or (3) due to patient preference [2, 4, 5]. Primary pharmacological therapy may also be appropriate if there are clinical contraindications to surgery or experienced pituitary neurosurgeons are not available. The current article provides a brief overview of the role of primary pharmacological therapy in the treatment of acromegaly in the context of Latin America and supports this with a representative case study.

### Current recommendations for primary pharmacological therapy

Surgical resection–usually, transsphenoidal microscopic or endoscopic surgery (TSS)–is recommended as the primary treatment of choice in patients with microadenomas or intrasellar macroadenmas, provided an experienced pituitary neurosurgeon is available to perform the procedure [3–6]. In these situations, surgery can lead to durable clinical, biochemical and tumor volume control of the patient [7]. In addition, TSS is indicated in patients with invasive macroadenoma associated with considerable mass effects such as rapidly progressing optic chiasm compression [4, 7]. Pharmacological therapy is generally used as adjuvant treatment in the setting of persistent disease despite surgical intervention [4, 8].

A role for primary pharmacological therapy, especially with SSAs, has been suggested in patients with macroadenomas with stable local mass effects who have a minimal chance of surgical cure (because of extrasellar extension of the tumor, especially into the cavernous sinus) or in patients who are poor surgical candidates or who express a preference for pharmacological treatment [3, 4, 6]. In patients with macroadenomas that are not likely to be cured with surgical intervention, debulking surgery may be recommended to improve the response to subsequent pharmacological therapy [4]. Radiotherapy is usually reserved as a third-line option for patients with recurrent or persistent disease activity after unsuccessful surgery, and who are resistant or intolerant to pharmacological treatment [8].

### Options for primary pharmacological therapy

Currently, long-acting SSAs are generally considered the first choice for the pharmacological treatment of acromegaly [2, 9, 10]. Long-acting formulations are more widely used due to increased compliance and patient convenience [2]. In approximately 50 % of cases where SSA primary therapy is used in clinical practice, the indication is the presence of a macroadenoma with lateral extension with or without cavernous sinus invasion, with the remainder of cases being for macroadenoma without compressive symptoms, for elderly patients, or based on patient preference [10]. The FDA approved subcutaneous octreotide in 1988, shortly after it was launched in Europe; it became available in Latin America in the early 1990s. Octreotide LAR was introduced in Europe in 1997 and was eventually approved by most Latin American Countries between 2000 and 2003. The most commonly used 20 mg formulation has an approximate monthly cost that ranges from $1000 USD for government institutions that usually purchase large amounts of the medication, to up to $2000 USD for the individual private patient. Lanreotide autogel became available in several Latin American Countries as of 2008. The government price for the 90 and 120 mg formulations is approximately $900 and $1200 USD, respectively. Lanreotide autogel’s relatively easy administration can result in significant indirect cost savings, since the patient does not need to come monthly to a specialized facility in order to get his/her injection.

A meta-analysis of prospective clinical trials up to the year 2003, suggests that long-acting SSAs reduce GH levels to <2.5 μg/L in just over 50 % of patients and normalize IGF-1 levels in a similar proportion [2, 11]. However, it should be noted that these figures are derived mainly from studies with a pre-selection bias since, for the most part, they involved Caucasian patients previously responding to non-depot formulations of SSAs. As expected, response rates are lower among non-preselected patients [2]. In a more recent multinational study looking at SSAs solely in unselected treatment-naïve patients, including participants from Latin America, a GH level ≤ 2.5 μg/L was achieved in 43–44 % and a normal age-adjusted IGF-1 in 34–38 % of patients (after 24 or 48 weeks) and both goals were achieved in 25–27 % [12]. Other longer-term studies in European, treatment-naïve populations have reported higher response rates for both GH (69–100 %) and IGF-1 (70–98 %), but these are likely to have been influenced by pre-selection bias and the use of per protocol rather than intention-to-treat analyses [13, 14]. To date, several predictors of biochemical response to SSAs have been identified, including gender, age, initial GH and IGF-I levels, and tumor mass, as well as adequate expression of somatostatin receptor types 2 and 5 [15, 16].

The SSAs are also effective at improving clinical symptoms of acromegaly, such as headache, fatigue, perspiration, and joint pain [12, 17], and have been shown to reduce the prevalence and severity of several measures of cardiomyopathy (arrhythmias, left ventricular hypertrophy, diastolic dysfunction and systolic dysfunction) and to improve certain cardiovascular risk factors, including hypertension and hypertriglyceridemia [14, 18, 19]. Interestingly, improvements in systolic function appear to be greatest when SSAs are used as primary therapy rather than after surgery, and it has been suggested that this may relate to a direct effect on the heart and/or better preservation of anterior pituitary function [18, 19]. Furthermore, SSAs induce clinically significant tumor shrinkage in close to 70 % of primarily treated patients, and some evidence suggests that this rate is increased when using this analogs for long periods of time [12, 14, 20–22]. Dose optimization with SSAs has been shown to be an effective means to improve treatment outcomes in patients with acromegaly who have inadequate response to the starting dose or who fail to achieve complete control of their disease [12, 23, 24].

Dopamine agonists (principally cabergoline) may also be used as primary pharmacological therapy [4, 10, 25]. Cabergoline has the advantage of oral administration and lower cost and may be the only available option if local resources are limited [25, 26]. In a meta-analysis of 9 studies from the literature, cabergoline alone was able to normalize IGF1 in 34 % of patients [26]. The evidence does not support the commonly held view that dopamine agonists, which also suppress the secretion of prolactin, are more effective in patients with hyperprolactinemia (who may harbor mixed GH- and prolactin-secreting pituitary tumors) [25, 26]. Combination therapy with SSAs and dopamine agonists may be appropriate in cases of partial response to monotherapy [4, 26].

Pharmacological therapy with the GH antagonist pegvisomant is generally restricted to patients with inadequate response or tolerability to SSAs [4]. Although initial studies reported a close to 100 % IGF-1 normalization rate, long-term studies reveal that this figure is between 60 and 70 % [27–29]. The addition of pegvisomant 10–30 mg/day is another option that has proven to be effective in patients resistant to monthly injections of SSAs [30]. Weekly and twice-weekly dosing (median dose 60 mg/week) has also been shown to be effective and could reduce costs *versus* the currently approved daily dosing regimen [31–33]. Pegvisomant is used, albeit as a tertiary option, in some (Venezuela, Argentina, Brazil) but not all Latin American Countries (Mexico, Chile, Colombia, Peru, Uruguay). Even at the lowest possible dose, the economic burden that represents the long-term treatment with pegvisomant can be overwhelming; the cost per month for a patient controlled on 10 mg a day is between $7000 and $9000 USD.

### The combination treatment approach

Rather than waiting for surgical approaches to fail before considering pharmacological therapy, another approach is the upfront consideration of pharmacological and surgical approaches together. Treatment with SSAs before TSS has been shown to improve surgical cure rates in patients with acromegaly [33–38]. However, not all studies have shown a benefit [39–42], and the case for pre-surgical treatment with SSAs continues to be debated [43, 44]. Similarly, as noted above, surgical debulking (in patients not initially amenable to complete surgical resection) has also been shown to improve the likelihood of biochemical control by SSAs [45–49].

Such combination approaches may be particularly appropriate for patients with advanced disease who may not be immediate candidates for surgery [4]. In addition to reducing tumor size and improving the chances of successful surgical resection, pre-surgical treatment with SSAs, by virtue of improving soft tissue swelling and achieving control of co-morbidities (such as hypertension, cardiomyopathy and diabetes), may aid in reducing cardiopulmonary anesthetic risk and facilitate intubation (Fig. 1).

**Table:** Case study: Successful primary pharmacological therapy for a tumor with optic chiasm compression and limited chance of surgical cure (Source: Ana Laura Espinosa de los Monteros, MD)

20 year old male
Progressive enlargement of hands and feet, as well as coarsening of facial features since age 15
2 years prior to consultation he developed headaches, fatigue and loud snoring
For the past 4 months he reports lack of morning erections and decreased libido
No significant past medical history. He had progressed through puberty unremarkably. No known allergies
Junior college student, actively engaged in sports. Does not smoke; no history of substance abuse
Unremarkable family history
*Physical examination*
Pulse 67 bpm, BP 134/76 mmHg, Weight 103 kg, Height 1.83 m, BMI 30 kg/m^2^
In no acute distress, evident acromegaloid features
Enlarged supracilliary arches, prognathism, macroglossia, enlarged hands and feet
Oily and thick skin, skin tags over anterior chest; no gynecomastia. Normal cardiopulmonary exam. No thyroid enlargement, no palpable lymph nodes; no palpable spleen or liver on abdominal examination. Gonadal exam appropriate for age and gender
Normal visual fields by confrontation (confirmed by Goldmann and automated perimetry), isochoric pupils, reacting normally to light and accommodation; normal extraocular muscle movement; normal fundi
*Hormonal evaluation*
GH nadir during OGTT = 5.3 μg/L (2-h glucose = 6.1 mmol/L [110 mg/dL])
IGF-1: 1,110 μg/L (3.5 × ULN)
Prolactin: 191 nmol/L (4,400 μg/L [normal 3–20 μg/L])
LH: 0.1 IU/L (normal 1.7–8.6 IU/L)
FSH: 0.3 IU/L (normal 1.5–12.4 IU/L)
Testosterone: 4.3 nmol/L (125 ng/dL [normal 280–800 ng/dL])
TSH: 0.3 mIU/L (normal 0.4–4.9 mIU/L)
Free T4: 5.1 pmol/L (0.4 ng/dL [normal 0.8–1.4 ng/dL])
Cortisol (8 AM): 254 nmol/L (9.2 μg/dL [normal 4.3–22.4 μg/dL])
*Pre*-*treatment MRI*
Gadolinium-enhanced, T1-weighted MRI, coronal view (Fig. 1)
5.5 cm, hyperintense mass with a small cystic component
Left parasellar extension with cavernous sinus invasion
Suprasellar extension with optic chiasm compression
*Treatment and course*
SSA q. 4 weeks, cabergoline 1.5 mg q. week
Levothyroxine 100 μg QD, hydrocortisone 10 mg BID
Remarkable clinical improvement, persistence of sexual dysfunction
*6* *month follow*-*up*
GH: 1 μg/L
IGF-1: 365 μg/L (1.15 × ULN)
Prolactin: 304 pmol/L (7 μg/L)
LH: 0.5 IU/L; FSH: 1 IU/L; testosterone: 6.9 nmol/L (200 ng/dL)
Serum cortisol 83 nmol/L (3 μg/dL; after 5 days of withholding hydrocortisone)
*MRI after 6* *months of treatment*
Gadolinium-enhanced, T1-weighted MRI, coronal view (Fig. 1) 90 % reduction of tumor, herniation of optic chiasm, aracnoidocele

## Case discussion

This is a case of acromegaly due to a giant, mixed GH- and prolactin-secreting adenoma. In spite of cavernous sinus invasion and optic chiasm compression, the patient did not appear to have any significant mass effects from the tumor. The patient had a striking response to pharmacological treatment with combined SSA and dopamine agonist therapy in terms of GH/IGF-1 and prolactin normalization, as well as reduction in tumor volume. Upon presentation, this patient had a minimal chance of surgical cure due to supra and parasellar involvement (with cavernous sinus invasion) of the pituitary macroadenoma, and thus represented a good candidate for primary pharmacological therapy in line with current acromegaly treatment guidelines and consensus recommendations [3, 4, 6, 50]. Upon long-term follow up, this young man was progressively able to first discontinue the dopamine agonist and, later-on, to reduce the dose of the SSA so after 3 years, he only required 20 mg of octreotide LAR every 2 months to maintain a GH of around 1 μg/L and an IGF-1 between 0.8 and 1.1 × ULN. This patient’s increased responsiveness to the SSA as time went by is a well-established observation [13] and undoubtedly has an important economic impact since the cost of therapy in the individual patient is significantly lowered. Considering that the adenoma almost disappeared with pharmacological therapy and the relatively low doses of SSA required by the patient to achieve biochemical control, we decided against submitting him to secondary management with either surgery or radiation therapy. Should this patient not have achieved biochemical and tumoral remission with pharmacological management, radiation therapy would have been an adequate option. Both conformal external beam radiation therapy [52] and radiosurgery [53] have proved to be effective and low-cost alternatives and undoubtedly are useful tools in the multi-modal strategy.

Unfortunately, presentation with large, often inoperable tumors is encountered frequently in Latin America due to delays in diagnosis and this can limit the options offered to the patient, irrespective of the available resources [3]. Nevertheless, this case provides a good example of how primary pharmacological therapy (in this instance using the combination of an SSA and a dopamine agonist) can be used to provide disease control in a patient with acromegaly who is initially not suitable for surgery. A major drawback of SSA is the need for long-term, indefinite therapy. In this regard, a substantial proportion of well-controlled patients can progressively increase their injection interval and a small but non-negligible number can eventually discontinue the drug [54].

## Conclusions

Primary pharmacological therapy may be the only viable option for many patients with acromegaly, especially those presenting with advanced disease with large inoperable tumors, as often occurs in Latin America [3]. In countries with limited resources, primary pharmacological therapy may also offer opportunities for disease control during delays in referrals for surgery. Many patients on primary pharmacological treatment will achieve good levels of biochemical control over the long-term, as well as decreased tumor size, improvements in symptoms and a reduction in severity of comorbidities.

The current evidence suggests that only a quarter of patients receiving SSA therapy for at least a year will not achieve any significant improvement in GH and/or IGF-1 levels or a reduction in tumor size [15]. Although a SSA is generally the drug of choice for first-line pharmacotherapy in acromegaly, in resource-poor regions, options may often be limited to cheaper, less effective drug classes. For instance, cabergoline may also be used as primary pharmacological therapy, especially if access to SSAs is limited.

Although access to SSAs has improved in Latin America, the costs of pharmacological therapy remain an important issue and it is worth considering opportunities for improving the cost-effectiveness of SSA therapy 49]. For instance, strategies that proactively utilize pharmacological therapies in conjunction with surgery offer opportunities for improved rates of biochemical control and cure [36, 49]. Furthermore, the use of drug combination therapy and/or extended dosing intervals may also help to improve response rates and reduce SSA doses [49, 51]. Thus, although improved access to recommended therapies would be the ideal solution, Latin American clinicians may need a flexible approach in order to maximize the benefits of primary pharmacological therapy for acromegaly within their budget constraints and local access to drug resources.
